# Using phage display selected antibodies to dissect microbiomes for complete de novo genome sequencing of low abundance microbes

**DOI:** 10.1186/1471-2180-13-270

**Published:** 2013-11-27

**Authors:** Devin W Close, Fortunato Ferrara, Armand EK Dichosa, Sandeep Kumar, Ashlynn R Daughton, Hajnalka E Daligault, Krista G Reitenga, Nileena Velappan, Timothy C Sanchez, Srinivas Iyer, Csaba Kiss, Cliff S Han, Andrew RM Bradbury

**Affiliations:** 1 Bioscience Division, Los Alamos National Laboratory, Los Alamos, NM, USA; 2 New Mexico Consortium, Los Alamos, NM, USA

**Keywords:** Phage antibodies, Genome completion, Single cell genomics, MDA, Flow cytometry

## Abstract

**Background:**

Single cell genomics has revolutionized microbial sequencing, but complete coverage of genomes in complex microbiomes is imperfect due to enormous variation in organismal abundance and amplification bias. Empirical methods that complement rapidly improving bioinformatic tools will improve characterization of microbiomes and facilitate better genome coverage for low abundance microbes.

**Methods:**

We describe a new approach to sequencing individual species from microbiomes that combines antibody phage display against intact bacteria with fluorescence activated cell sorting (FACS). Single chain (scFv) antibodies are selected using phage display against a bacteria or microbial community, resulting in species-specific antibodies that can be used in FACS for relative quantification of an organism in a community, as well as enrichment or depletion prior to genome sequencing.

**Results:**

We selected antibodies against *Lactobacillus acidophilus* and demonstrate a FACS-based approach for identification and enrichment of the organism from both laboratory-cultured and commercially derived bacterial mixtures. The ability to selectively enrich for *L. acidophilus* when it is present at a very low abundance (<0.2%) leads to complete (>99.8%) *de novo* genome coverage whereas the standard single-cell sequencing approach is incomplete (<68%). We show that specific antibodies can be selected against *L. acidophilus* when the monoculture is used as antigen as well as when a community of 10 closely related species is used demonstrating that in principal antibodies can be generated against individual organisms within microbial communities.

**Conclusions:**

The approach presented here demonstrates that phage-selected antibodies against bacteria enable identification, enrichment of rare species, and depletion of abundant organisms making it tractable to virtually any microbe or microbial community. Combining antibody specificity with FACS provides a new approach for characterizing and manipulating microbial communities prior to genome sequencing.

## Background

Microbes are critical symbiotes for humans, where upwards of 100 trillion foreign cells from more than 1000 different species reside [1,2]. The gut is host to the bulk of the microflora, where bacteria are the most abundant, outnumbering eukaryotes and viruses by orders of magnitude. While a handful are known human pathogens, the majority of these bacteria, such as *Lactobacillus* sp. are commensal or mutualistic, exerting their influence through probiotic functions [3]. Studies in mice and humans implicate gut bacterial influence not just in digestion of nutrients [3], but in fat storage [4], modulation of bone-mass density [5], angiogenesis [6], protection against pathogens [7], and immune functions [8,9]. Conditions such as Crohn’s disease [10], diabetes [11,12], and obesity [13-15] have all been directly linked to an imbalance of gut microflora. Despite an explosion of research in recent years, the ecology and mechanistic details of complex microbiomes such as those found in the gut remain enigmatic, and new methodologies for dissection and characterization are needed.

Metagenomics refers to a powerful set of genomic and bioinformatic tools used to study the diversity, function, and physiology of complex microbial populations [16]. Substantial advances in microbiome research have been driven by the extensive use of next generation sequencing (NGS) technologies, which allow annotation and characterization of microbiomes using targeted (e.g. hypervariable regions of 16S rRNA [17]) or shotgun approaches [18]. Targeted approaches are suboptimal in the identification of low abundant species [18], and even though identification of most species from a population is possible using shotgun sequencing, assembly of complete genomes of individual species is rarely possible unless those species are highly abundant. Moreover, as complexity increases, dataset resolution decreases, reducing the ability to comprehensively analyze community structure. Recent reports provide promising advances in metagenomic binning and assembly for the reconstruction of complete or near-complete genomes of rare (<1%) community members from metagenomes. Albertesen et al. [19] have described differential-coverage binning as a method for providing sample-specific genome catalogs, while Wrighton et al. [20] have also been successful in sequencing more than 90% of the species in microbial communities. In another approach, either GC content [21] or tetranucleotide frequency [20] combined with genome coverage patterns across different sample preparations was used to bin sequences into separate populations, which were then assembled under the assumption that nucleotide (or tetranucleotide) frequencies are constant for any specific genome. Sequencing throughput is continually improving and is expected to provide access to increasingly lower abundance populations and improvements in read length and quality will reduce the impact of co-assembly of closely related strains (strain heterogeneity) on the initial *de novo* assembly. While these approaches represent exciting advances in bioinformatic tools, experimental tools for reducing the complexity of a population prior to sequencing, such as enriching for low abundant organisms or intact cells, provide alternative and complementary approaches to improve genomic analysis of such complex systems [22].

A variety of experimental methods have been used to decrease sample complexity prior to sequencing. The most commonly used tool for decreasing sample complexity is probably single cell genomics (SCG) [23,24] which utilizes flow cytometry, microfluidics, or micromanipulation to isolate single cells as templates for whole genome amplification by multiple displacement amplification (MDA) [25-27]. As it requires only a single template genome, it allows the sequencing of “uncultivable” organisms. For example, a recent paper from the Quake group used microfluidics to isolate single bacterial cells from a complex microbial community, using morphology as discriminant, before genome amplification and analysis [28]. SCG approaches rely on MDA, and while MDA can generate micrograms of genomic amplicons for sequencing from a single cell, amplification bias, leading to incomplete genome coverage, is a major inherent limitation [29,30]. In fact, a recent survey of 201 genomes sequenced from single cells had a mean coverage of approximately 40% [31]. A clever use of single amplified genome (SAGs) assembly improved coverage to >90% for 7 of the 201 genomes, with mean coverage being approximately 70% for the 21 genomes when assembled from multiple SAGs. MDA-associated Amplification bias has been improved for eukaryotic cells using a technique called MALBAC [32], but these improvements have yet to be shown for prokaryotic genomes and still rely on random, or morphologically based, cell sorting. Such random sorting of single microbial cells from complex mixtures is expected to bias against rare species and may require sorting and sequencing of hundreds to thousands of cells before a rare genome can be obtained.

Increased input template number can overcome MDA amplification bias, or difficulties in processing and sorting single cells from biofilms, and provide near complete genome coverage. Potential methods for accomplishing this include inducing artificial polyploidy or using gel microdroplets [24,33]. However, in both of these cases, rare species may still be missed if sufficient numbers of single cells cannot be sorted. This has been partially addressed in a recently published “mini-metagenomics” approach. MDA product coverage was improved by creating bacterial pools by flow cytometry, with ~100 bacteria in each pool. Screening of these pools for 16S rDNA sequences of the bacterial species of interest, followed by deep sequencing of the positive pools, allowed assembly of a relatively complete genome from different pools containing the same 16S RNA sequences [34].

An alternative approach to simultaneously address both amplification bias and isolate rare species is to use antibodies recognizing specific microorganisms within microbial communities to enrich and/or subtract bacterial species prior to sequencing. We hypothesized that enrichment by selective sorting in this way could provide a powerful method for significantly increasing input template number to obtain complete genomes of low abundance species, akin to creating a small microbiome in which all members expressed a single target recognized by the antibody of interest.

In the present work, we developed a selection and screening pipeline using phage display and flow cytometry to isolate a single chain Fv (scFv) antibody that can: i) identify a bacterial species, *Lactobacillus acidophilus*, with extreme specificity; and ii) be applied to a microbiome, using fluorescence activated cell sorting (FACS), to identify, enrich, and deplete targeted species from bacterial mixtures. We further demonstrated that if this approach was applied to a mock community containing *L. acidophilus*, rather than the pure single species, antibodies recognizing *L. acidophilus* could be isolated. This phage display selection method is highly adaptable to recognition of any organism and provides a unique tool for dissection and sequencing of rare species from complex microbiomes.

## Results

### Selection against intact bacteria using phage display and screening by flow cytometry

**Figure 1:**
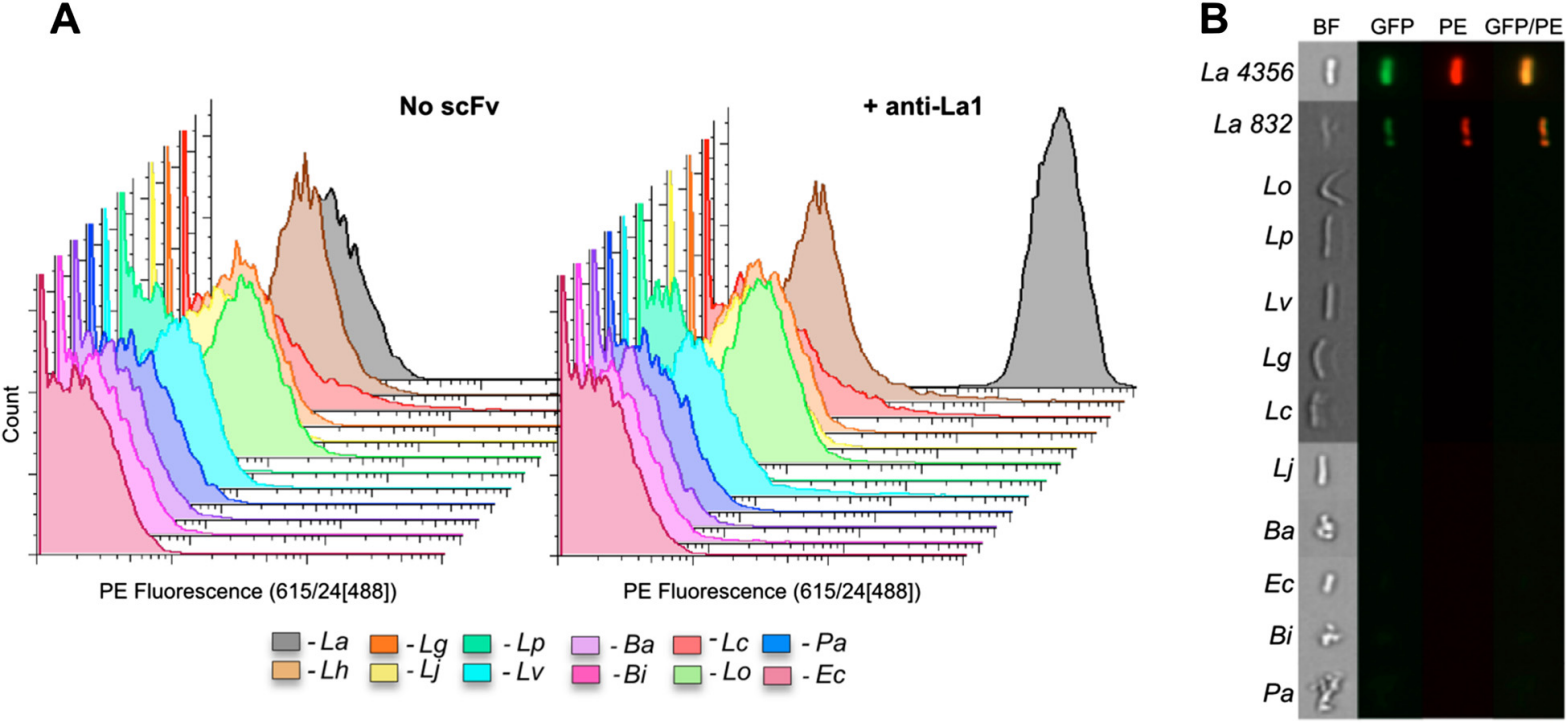
**A phage display derived single chain fragment (scFv) was selected that binds *Lactobacillus acidophilus *(L.a.) specifically.** Various bacterial species (see Table 1 for abbreviations) were mixed with the α-La scFv-SV5-GFP-s11 fusion protein and stained with α-SV5-IgG-PE and/or GFP1-10. Binding specificity was confirmed using both standard **(A)** and imaging **(B)** flow cytometry (BF = Bright Field, GFP = Green Fluorescent Protein, PE = Phycoerytherin).

We chose the probiotic *Lactobacillus acidophilus* ATCC 4356 as a target for our approach. *Lactobacilli* such as sp. *acidophilus* are widely studied gut microbes with probiotic functions including digestion, immune function, and prevention of diarrhea [35]. Antibody selections were performed against *L. acidophilus* using two methods. In the first, the bacteria were coated on Immunotubes (Nunc), while, in the second, selection was carried out by centrifugation. For each selection we used a previously described naïve scFv library displayed on M13 filamentous phage [36]. Two to three rounds of selection, with increasing stringency, were performed prior to re-cloning enriched scFvs into pEP-GFP11 [37] for screening. This vector generates scFv proteins in fusion with two different detection tags: SV5, recognized by a monoclonal antibody [38] and S11, a split green fluorescent protein (GFP) tag that fluoresces when complemented with GFP1-10 [39]. The simultaneous use of both tags enhances signal-to-noise ratio when testing putative clones for binding activity against *L. acidophilus* in flow cytometry. ScFv culture supernatant was incubated with *L. acidophilus* followed by staining and the *L. acidophilus* bacteria analyzed using an LSRII flow cytometer (Becton Dickinson). Sequencing revealed one unique scFv (α-La1) from the immunotube selection, and three unique scFvs (α-La2, α-La3, and α-La4) from the selection by centrifugation (Additional file 1). The α-La1 scFv was found to be highly specific for *L. acidophilus*, binding to all tested *L. acidophilus* strains (ATCC strains 4356 and 832), but not to a panel of other gut bacteria, including *Bifidobacterium* sp., *Peptoniphilus* sp., *E. coli*, and six different species of *Lactobacillus* (Figure 1 and Table 1). Our analysis included *Lactobacillus helveticus*, the closest species to *L. acidophilus*, the 16S rRNA sequence of which shares >98% identity [40]. The other three α-La scFvs showed similar degrees of specificity. We proceeded with the α-La1 scFv for the remainder of the study due to greater expression and apparent affinity relative to the other α-La scFvs (Additional file 2). The specificity of the α-La1 scFv was also further validated using the AMNIS Image-Stream Mark II flow cytometer (Amnis Corporation), which captures microscope images in a flow cytometric configuration (Figure 1B).

**Table 1:** Bacterial species used in this study

**Organism**	**ATCC strain ID**
*Lactobacillus acidophilus (La)*	4356
*Lactobacillus acidophilus* (*La)*	832
*Lactobacillus helveticus (Lh)*	521
*Lactobacillus parafaringis (Lp)*	F0439
*Lactobacillus oris (Lo)*	F0423
*Lactobacillus vaginalis (Lv)*	EX336960VCO5
*Lactobacillus gasseri (Lg)*	JV-V03
*Lactobacillus crispatus (Lc)*	JV-V01
*Lactobacillus johnsoni (Lj)*	332
*Bifidumbacterium adolescentis (Ba)*	15703
*Bifidumbacterium infantis (Bi)*	15697
*Bifidumbacterium infantis spp. longum (Bl)*	15707
*Peptoniphilus asaccharolyticus (Pa)*	29743
*Escherichia coli (Ec)*	4157

Lactobacillus strains were grown in ATCC No. 416 *Lactobacilli* MRS broth.

All other strains were grown in ATCC No. 1053 Reinforced Clostridial broth with the exception of Ec which was grown in Luria Broth.

**Figure 2:**
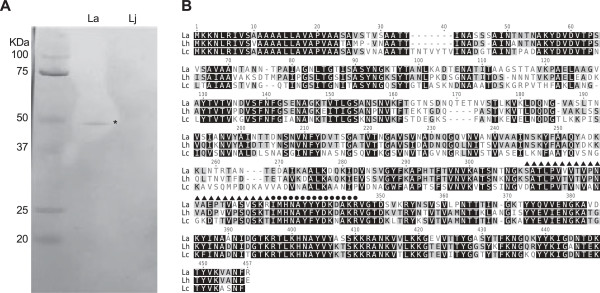
**The antigen recognized by the α-La scFv is the S-layer protein A. A)** Western blot using α-La scFv as primary antibody and α-SV5-Alkaline Phosphatase as secondary for detection. An obvious ~45KDa band appeared in the lane containing *L. acidophilus* (La) lysate and not the lane containing *L. johnsonii* (Lj) lysate was extracted and identified using MS/MS. **B)** Protein alignment of S-layer proteins from closely related Lactobacillus species (La = *Lactobacillus acidophilus*, Lh = *Lactobacillus helveticus*, Lo = *Lactobacillus oris*). The two La peptide sequences recovered after MS/MS analysis are indicated with solid triangles or circles above the sequence.

The specific surface antigen recognized by all the α-La scFvs was identified as the *L. acidophilus* S-layer A protein, (SlpA; Uniprot P35829) using western blotting and mass spectrometry (Figure 2). SlpA proteins are highly abundant, paracrystalline surface glycoproteins that make obvious targets for scFv recognition [41,42]. Further analysis following deglycosylation of the bacterium revealed that recognition was not mediated by glycosylation of the protein (data not shown).

### scFv specificity to L. acidophilus in a mock community

**Figure 3:**
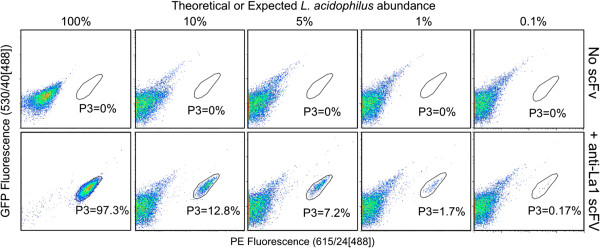
**The α-La1 scFv can identify *L. acidophilus *(La) specifically in a mixture of different species.** A “mock community” of 10 species where La was added at varying percentages (expected abundance). The percent La observed in each of the communities (gate P3) closely matched the expected La abundance.

We tested the use of the isolated α-La1 scFv protein to detect varying abundances of *L. acidophilus* within a mixture of different bacterial species. We individually grew a total of ten species in their respective growth media (Table 1). The various species were mixed to generate a “mock” community, which enabled us to control the relative composition of different species within the mixture. All species in the mock community were added at equal concentrations (see Methods). The four resultant mock communities contained 10% of each of these species, and differed only in their relative abundance of *L. acidophilus* at 10%, 5%, 1%, and 0.1% in the community. Staining with purified α-La scFv was followed by analysis by flow cytometry. Pure *L. acidophilus* stained with α-La1 scFv was used to establish the *L. acidophilus* analysis gate (P3; Figure 3) as reference for varied *L. acidophilus* abundances in the mock communities. Ten thousand events from each mock community were analyzed. We observed 12.8%, 7.2%, 1.7%, and 0.17% *L. acidophilus* in the mock 10%, 5%, 1%, and 0.1% communities, respectively. This degree of accuracy supports the possibility that the scFv can detect target bacteria within a population, with abundance less than 0.2%, and further supports the specific nature of the α-La1 scFv.

### Targeted enrichment of single L. acidophilus cells from yogurt microbial community

**Figure 4:**
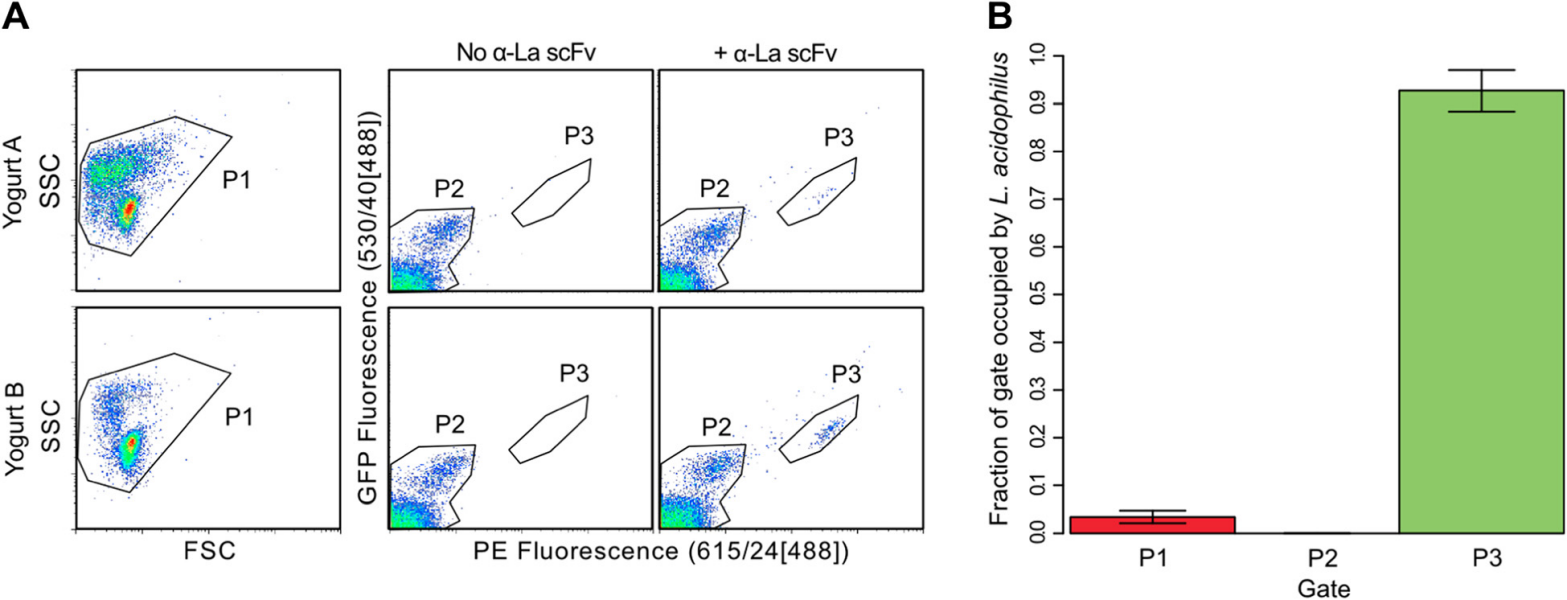
**Identification of *L. acidophilus *(La) in a mixture of bacteria extracted from yogurt. A)** La was identified in different bacterial extractions only when the α-La1 scFv is used in the staining. Single or multiple cells were sorted using pre-sort (P1), negatively sorted (P2) and positively sorted (P3) gates. **B)** 16 s rRNA sequencing of single cells sorted from all three gates revealed significant enrichment of *L. acidophilus* from an average of 3.4% (95% *CI*: 2.1-4.8%) in the pre-sort (P1) community to 90.6% (95% *CI*: 86.6-94.6%) in P3 (n = 4, p-value <2.2x10^-16^ when using a standard Chi-squared test).

The ability to sort single *L. acidophilus* cells using the α-La1 scFv was subsequently tested on cultured yogurt, a natural, heterologous community the constituents of which are reported to include *Streptococcus thermophilus, Lactobacillus delbrueckii* Subsp*. bulgaricus, Lactobacillus delbrueckii* Subsp*. lactis, Lactobacillus acidophilus,* and *Bifidobacterium lactis.* Our aim was to validate specificity and test the ability of our selected scFv to recognize *L. acidophilus* from a culture even though the scFv was selected against bacteria grown in the laboratory. Bacteria were isolated using methods previously described based on a series of density gradient centrifugations to remove sample debris prior to bacterial cell isolation [33]. After staining with α-La1 scFv-GFP + α-SV5-PE (phycoerythrin), 0.1-5% of the total population, depending upon the yogurt preparation, fell into the *L. acidophilus*-specific gate (gate P3) (Figure 4A). Single bacterial cells were sorted from the pre-sort (P1), negatively sorted (P2), and positively sorted (P3) gates for amplification by MDA and subsequent 16S rDNA sequencing. We identified the species origin of 244 individual cells sorted from four different replicates (Additional file 3). The dominant species in the community was *Streptococcus thermophillus*, with *Lactobacillus delbruekii* and at least eight other species identified, including species that were not expected to be found in the yogurt culture. On average, sequencing showed *L. acidophilus* recovery at 3.4% (95% *CI*: 2.1-4.8%) in the pre-sort (P1) community, enrichment at 90.6% (95% *CI*: 86.6-94.6%) in P3, and complete absence in P2 (Figure 4B), thereby demonstrating the feasibility of species depletion. In three of the replicates, *L. acidophilus* sequence was not observed in the pre-sort (P1) sample (Additional file 3), but was nevertheless enriched and identified in the P3 gate, indicating that the *L. acidophilus* likely would not have been identified using standard single cell sorting and analysis.

### Obtaining a complete genome using scFv targeted enrichment

**Figure 5:**
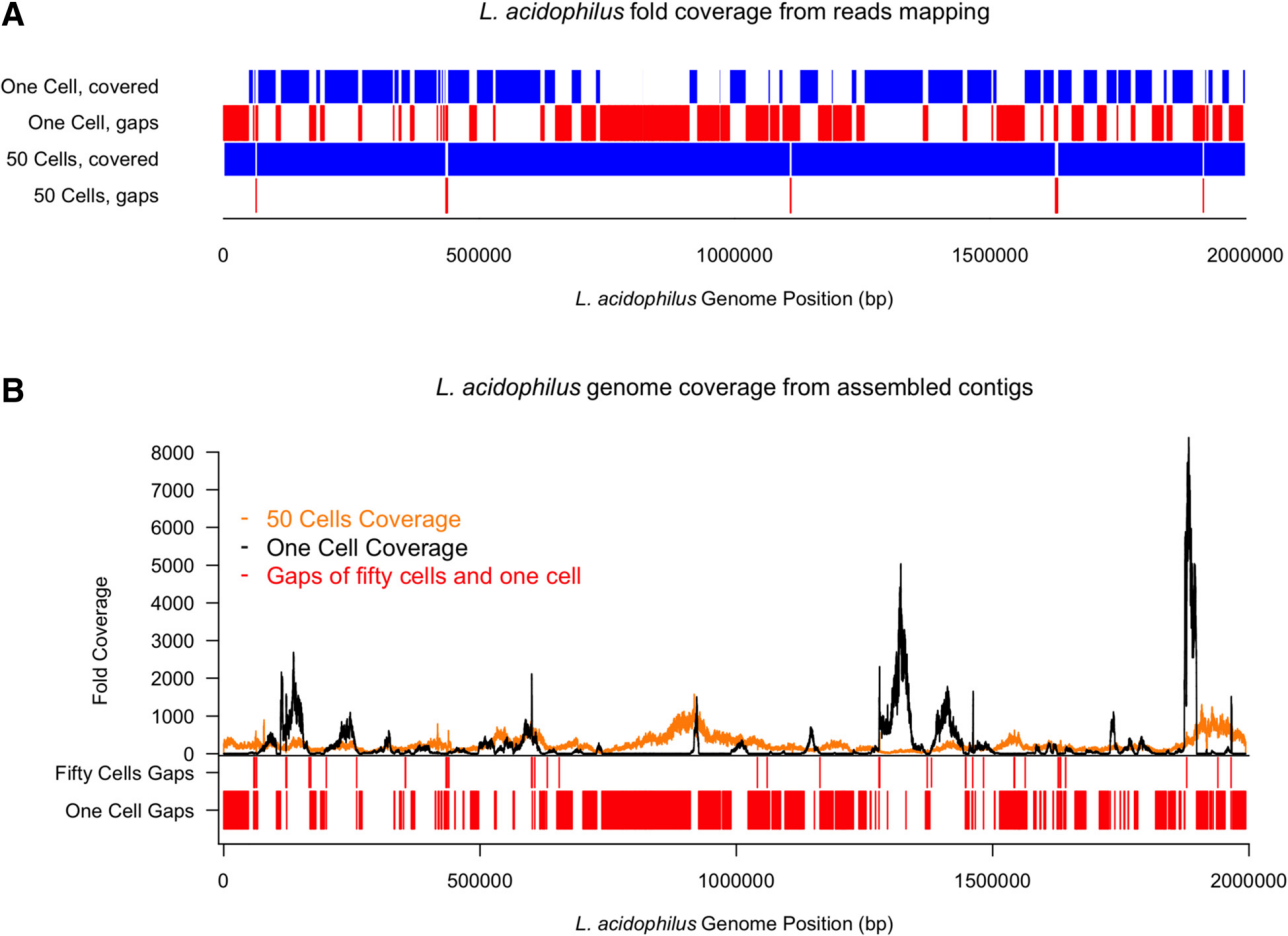
**Enrichment of genomic DNA using the α-La1 scFv significantly improves genome coverage and amplification bias.** A single cell per well, or 50 cells per well were sorted from gate P3 and sequenced using Illumina MiSeq. **A)** Sequencing reads mapped to *L. acidophilus* NCFM shows significantly more complete coverage (99.8%) when using the 50-cell template versus a single cell template. **B)***De novo* assembled contigs mapped back to the reference sequence show essentially complete coverage (>99.8%) with far less amplification bias.

One of the primary goals of this study was to show that targeted enrichment of template using phage derived antibodies and FACS can be used to generate complete genome sequences of rare species, with the specificity conferred by the selected scFv enabling the enrichment of enough template to complete a genome without any further downstream cultivation or chemical treatment prior to MDA. To test this idea, *L. acidophilus* was sorted from one of the bacterial yogurt extractions, (*L. acidophilus* abundance <0.2% by flow analysis) as either single cell or 50-cell templates for MDA, and sequenced using the Illumina MiSeq platform. For reference mapping, reads from both the single and 50-cell sorted amplicons were normalized and mapped to *L. acidophilus* NCFM (Figure 5). In parallel, as reference genomes are unavailable in most cases, we also assembled the genome *de novo* using the normalized reads. The assembly tool CLC was used to both map reads and assemble contigs *de novo*. Having a reference genome available allowed us to accurately assess the extent of genome coverage using both mapped reads and *de novo* assembly. As we hypothesized, reads mapping from the 50-cell template yielded near-complete genome coverage at 99.9%, while the single cell template fell short at 68% with far more amplification bias (Figure 5). Bias is clear (Figure 5B) in the single cell template with a large portion of the genome lacking coverage while other regions are covered at very high frequencies of >8,000 fold. For the *de novo* assembled genome, the 50-cell template yielded 124 contigs (compared to 555 for the single cell) with >99.8% coverage of the reference and ~8-10% contamination by sequences from non-*L. acidophilus* species. The contaminating non-*Lactobacillus* reads were identified by searching assembled contigs in sequenced microbial genomes. We found that the single cell data was contaminated with sequences from bacteria close to a sequenced *Pseudomonas* genome (accession number, CP002290) and the 50-cell data was contaminated with genomic sequences related to *Rhodopseudomonas* (CP000283), *Bradyrhizobium* (BA000040) and *Nitrobacter* (CP000115). 13.37% of the single cell read data mapped to the *Pseudomonas* genome and 3.23% of the 50-cell data mapped to the *Rhodopseudomonas* genome, 0.6% to the *Bradyrhizobium* and 0.14% to the *Nitrobacter*. The contaminations were likely generated during the cell sorting and/or the MDA process. MDA-related contaminants, such as non-specific amplification and DNA presented in reagents, are common to virtually any approach that utilizes whole genome amplification [33,43-46]. Beside possible contamination from the MDA process, most contaminants were probably introduced during the cell sorting process since contaminated sequences were not shared between single and 50-cell results. We hypothesize that sorted specific cells may contain contaminating cells in the same droplet (even though we used the highest purity sorting setting), or that contaminating DNA, either free in solution or attached to the targeted cell may be sorted and become an MDA template. We believe it more likely that the *Rhodopseudomonas* genome, which was 34% covered, may have been introduced by cell contamination, while lower level contamination may have occurred via the second mechanism. Fortunately, the vast majority of contaminant reads was easily removed and did not interfere with full data analysis of assembled contigs. To assess coverage, *de novo* assembled contigs were mapped back to the reference and the resulting coverage was >99.8% for the 50-cell template and 63% for the single cell. These values are highly similar to those expected from draft coverage of cultured bacteria, indicating that template number enrichment using specific scFvs and FACS can be used to sequence very low abundance (and potentially uncultivable) genomes in a community once a specific antibody is available.

### Selecting antibodies against a mock community

To determine whether this method can be applied to more complex microbial communities, we selected phage antibodies against the mock community used above, with each bacterial species present at ~10%. Selection was carried out by centrifugation, and after two rounds, the heavy chain complementarity determining region 3 (HCDR3) of the complete antibody output was sequenced by Ion Torrent. The HCDR3 is the most diverse CDR, contributes most to antibody binding specificity, and is widely used as a surrogate for VH and scFv identity [47-49]. Using the Antibody Mining ToolBox [50], the HCDR3s of the antibodies selected against the mock community were identified and ranked for abundance. As shown in Table 2, three of the twenty most abundant antibodies had HCDR3s that were identical to three of the previously selected antibodies (α-La2, α-La3, and α-La4) recognizing *L. acidophlius*, indicating that, in principle, it may be possible to select species specific antibodies directly against individual bacteria in complex bacterial communities, without the need to culture the individual bacteria. However, validation of this possibility will require additional experimentation and selection on natural microbiomes rather than the mock community used here.

**Table 2:** HCDR3 sequences enriched from selection against a mock community

**Rank**	**Unique HCDR3 sequence**	Number of reads*	**Frequency of reads**	***L. acidophilus *** **Binder**
1	CSTDDYGGNW	212506	17.7%	α-La2
2	CARAGRGTSYYGMDVW	142822	11.9%	
3	CARVGDGYNYAFDIW	34320	2.9%	
4	CAVAGTGYAFDIW	17429	1.4%	
5	CARAGGGTSYYGMDVW	11394	0.9%	
6	CAKLRGGPTKGDWYFDVW	9688	0.8%	
7	CATGDAFDMW	9287	0.8%	α-La3
8	CARGHYGMDVW	7675	0.6%	
9	CARDEGNAFDIW	7303	0.6%	
10	CARGSLGAFDIW	5761	0.5%	α-La4
11	CAKLRGPTLPRYSFDYW	5601	0.5%	
12	CARDPLGKLGPEEYYYGMDVW	4598	0.4%	
13	CARDSMWVVAAKRKLHNCFDPW	4939	0.4%	
14	CARDRGYGVDYW	3331	0.3%	
15	CARDLGAGMDVW	3256	0.3%	
16	CARQQLAAFDIW	3037	0.3%	
17	CARDKGHEAFDIW	2589	0.2%	
18	CARDGGDAFDIW	2029	0.2%	
19	CARDYGEAFDIW	1585	0.1%	
20	CARIGGGKRRSHFDYW	1438	0.1%	

*Total number of quality reads from the Ion Torrent sequencing run = 1,203,589.

## Discussion

The expanding field of metagenomics continues to search for robust ways to obtain high-quality genomes from under-represented or rare species in a given sample. Improvements in sequencing throughput will enable access to lower abundance populations, but a “pre-enrichment/pre-clearing” step before the analysis can provide complementary and significant results. We describe a novel and adaptable approach for sequencing low abundance genomes from microbial communities, with potential improvements in the genomic coverage of low abundance species where standard single cell approaches result in incomplete genomes or may have missed the organism altogether. We demonstrate the use of phage display to select antibodies against a bacterial species with exquisite specificity. The use of *in vitro* display potentially allows the method to be adapted to any organism or microbiome, does not rely on commercially available antibodies, and generates antibodies that are highly renewable and amenable to further engineering to modify affinity or specificity [51]. To demonstrate the feasibility of the approach, we first targeted *Lactobacillus acidophilus*, a bacteria naturally found in environmental samples from food to feces and is a principal commensal bacterium of the human gut. The tested α-La1 scFv proved to be extremely specific and did not recognize other common gut microflora (such as *Bifidumbacterium* and *E. coli*). While it is practically impossible to prove that this scFv does not recognize any other bacteria, when tested on other *Lactobacilli* such as *L. helveticus*, which is highly similar to *L. acidophilus*[40], we did not observe binding, providing strong evidence that the scFv is species-specific.

The target protein recognized by our scFv was identified as the Surface layer protein A (SlpA). S-layer proteins are highly abundant and ubiquitous crystalline surface structures [41,42] that have been implicated as a principal component for the organism’s probiotic functions [52,53]. Other Lactobacilli tested in this study produce S-layer proteins that are highly similar (73% identical for *L. helveticus*) (Figure 2B), but which can nevertheless be distinguished by our α-La1 scFv, demonstrating the high degree of specificity achievable. Since S-layer proteins are common to many bacteria, future work may involve re-engineering the α-La1 scFv to target S-layer proteins from other organisms, an option that is only possible with *in vitro* derived antibodies [51].

Coupling the specificity of phage-selected α-La1 scFv with FACS allowed precise manipulation of a population on a per-cell basis, making possible the sufficient enrichment of *L. acidophilus* for >99.8% genome coverage using both reference mapping and *de novo* assembly. While it is common to observe this level of coverage for *de novo* assembly when the target organism is cultured prior to sequencing in the laboratory, the level of coverage reported here for a bacteria extracted from an environmental sample is exceptional. For sequencing, we easily and rapidly sorted 50 *L. acidophilus* cells from an environmental sample (yogurt) where *L. acidophilus* comprised ~0.2% of the population and were able to rapidly detect and quantify *L. acidophilus* at ~0.1% in a mock community comprising nine other species. Although we only tested compositions as low as ~0.1%, we are confident that *L. acidophilus* could be identified from mixtures where it is even lower in relative abundance with detection limited solely by the total number of cells available in a mixture and time available for sorting.

While detection and enrichment of rare species is an obvious use of these antibodies, depletion of common species may be equally important, as bias towards high abundance species is a well-known issue when performing shotgun metagenomics [54-57] and, potentially, non-targeted single cell genomics. Our single cell analysis shows that *L. acidophilus* is completely depleted from the sample in the negative sort gate (P2; Figure 4), demonstrating the feasibility of both depletion and enrichment. Separation methods, namely immunoprecipitation, micromanipulation, and flow cytometry have been described to improve genome sequencing, and the approach described here may also be applicable to other microbes found in microbiomes without being limited to organisms with innate fluorescence [58], distinct morphology and/or high genome copy number [43].

In this study we generated a scFv against an organism that can be cultured in the lab as a demonstration that recombinant antibodies can be raised against a specific organism and used to dissect, phylotype, and recover complete genomes for organisms from microbial communities. We used an organism with a reference genome in order to accurately assess genome coverage. Future studies will involve selecting antibodies directly against uncultivable organisms within complex microbiomes. We provide proof of principle, using selection against a mock community, that such an approach is potentially feasible: HCDR3 sequences of three of the antibodies selected against the pure culture were identical to those of antibodies selected against the mock community. While this is promising, it is likely that selection procedures will have to be modified in order to select antibodies against the many different species present in a natural microbial community. In particular, we have previously shown that selection against a specific antigen is far more efficient when carried out against the individual antigen than when the antigen is present in a mixture of other antigens [59]. The situation is likely to be even more challenging for microbial communities, and may require selection in emulsions [60,61], microfluidics [62-64] or against individual cells [65,66] to ensure that individual bacteria are isolated from one another during the selection process. If the identity of the recognized bacteria in the microbiome is unimportant – i.e. the goal is to catalog genome sequences present in a microbiome, whatever they are – the use of this method may be relatively straightforward. It is likely to be more challenging, however, if the goal is to select antibodies against particular species in a population, unless an alternative means of bacterial isolation, such as fluorescent in situ hybridization [67], is available. One possible approach, which may be successful in microbiomes comprising few species, would be to select a panel of positive antibodies against different species within the community, and then deconvolute species recognition using FACS and deep sequencing in a manner similar to that described here, after antibody selection and sorting. However, the number of bacteria that can be extracted from environmental samples easily exceeds the number required for phage selection suggesting that this approach will be difficult for more complex populations. Since depletion is as feasible as enrichment using these scFvs with FACS, it may be possible to iterate the process using scFvs against high abundance species for their subtraction and, thus, enrich for the low abundance organisms. Even if antibodies cannot be raised to low abundance organisms, depletion of high abundance organisms in a mixture will concentrate the low abundance ones, and so lead to improved taxonomic identification and genome recovery.

The described approach also has potential not only for the genome sequencing of novel and uncultivable organisms, but also in comparative genomics. In this regard, selection of antibodies against organisms initially grown in the lab then used on environmental and clinical samples holds great potential for medicine and epidemiology [68,69]. For example, a recent study [46] reports the use of a commercially available IgG antibody for targeted enrichment using immunomagnetic separation (IMS) to fully sequence *Chlamydia trachomatis* directly from clinical isolates without culture. Our approach could extend on this work by adding a mechanism for the initial selection of suitable antibodies for studying pathogenic, probiotic, or other organisms. Near complete coverage, such as that provided by enrichment with phage-selected scFvs, is paramount for high resolution genomic comparisons. In fact, while a discussion of genome differences is outside the scope of this study, we observed at least 14 Single Nucleotide Polymophisms (SNPs) when comparing the extracted *L. acidophilus* to the reference genome showing that the α-La scFv reported here could be used immediately for future comparative genome studies on human-derived *L. acidophilus* for both research and clinical purposes.

## Conclusions

In this paper we demonstrate the power of combining phage antibody selection directly on bacteria with fluorescence activated cell sorting and deep sequencing to either enrich, or deplete, bacteria recognized by specific selected antibodies. Using this approach it becomes possible to assemble genomes directly from complex microbiomes without preculture, or to subtract recognized bacterial species from a microbiome to facilitate genomic analysis of the remaining species. This approach has potential to be applied to different species in different and complex microbial communities.

## Methods

### Bacterial cultures and media

*E.coli* DH5αF’ was used to propagate phage and *E.coli* BL21 Gold was used to express recombinant scFvs. *E. coli* was grown in 2xyT media containing 1% glucose at 37°C. During phage propagation, ampicillin and kanamycin were used final concentrations of 100 and 25 μg/μl, respectively. *Lactobacillus* spp. (Table 1) were grown in *Lactobacilli* MRS Broth (BD 288130) with 5% CO_2_ atmosphere at 37°C with shaking at 250 rpm. *Bifidumbacterium spp.* (Table 1) and *Peptoniphilus asaccharolyticus* were grown in Reinforced Clostridial Medium (BD 218081) with anaerobic condition (85% N2, 5% H2 and 10% CO2) at 37°C with shaking at 250 rpm. After growing for 18–24 hours, cells were washed twice by spinning down at 3000xg for 5 min, resuspension in 10 ml of washing buffer (WB = PBS, BSA 1%, 2 mM EDTA). After the final washing step cells were resuspended in PBS.

### Panning

A 10 ml overnight (ON) culture of *L. acidophilus* was grown and washed as described above. Cells were diluted in PBS to an OD_600_ of ~1.0 (approx. 10^9^ cells/ml) and used for immune-tube (Nunc) coating. The coating process consisted of 1 h incubation at 37°C followed by ON incubation at 4°C. The tube was then blocked with 2% skim milk PBS solution (MPBS) for two hours at room temperature (RT). Phage were generated as described previously and 10^12^ phage particles of our phage display library [36] were blocked for 1 h at RT with MPBS. Phages were then added to the bacteria coated immune-tube and rotated for 30 min at RT followed by 1.5 h standing at RT. Unbound phages were removed by washing the tube with increasing stringency (number of washes were 20, 25, 30 for the 1^st^, 2^nd^ and 3^rd^ round of selection respectively) with PBS containing 0.05% Tween (PBST) followed by the same number of washing steps with PBS. After the final wash phages were eluted adding 750 μl of 0.1 M HCl solution for 5 min at RT. The solution was then neutralized with 250 μl of 1.5 M Tris-base pH 8.8 solution. This was followed by phage propagation and titration as described in Sblattero et al. [36]. Panning by centrifugation was performed by incubating 10^9^ bacterial cells with 10^12^ phage particles, previously blocked with MPBS, in an 1.5 ml Eppendorf tube for 2 h at RT. Bacteria with bound phages were pelleted by spinning at 10000xg for 30s and supernatant containing unbound phages was removed. Bacteria with bound phages were further washed with PBST and PBS (5 and 10 each for 1^st^ and 2^nd^ rounds of selection, respectively) by resuspension in 1 ml of wash buffer and transfer to a new tube, followed by pelleting. Phages were eluted by resuspending the bacterial pellet after washes in 150 μl of 0.1 M HCl solution for 5 min at RT, and the solution was neutralized with 50 μl of 1.5 M Tris-base pH 8.8 solution. The resulting solution was pelleted and the supernatant containing phage particles was used for phage propagation and titration as described above.

### Screening

DNA encoding scFvs recovered from the third round selection output was cloned into the expression vector pEP-GFP11 [37]. The pEP-GFP11 vector expresses recombinant scFv protein in fusion with an N-terminal PelB leader and C-terminal SV5, 6x His, and GFP strand 11 tags. The DNA was digested with BssHII and NheI, purified, and ligated into the pEP-GFP11 vector. The ligation reaction was transformed into *E. coli* BL21 Gold electrocompetent cells, and positive clones were selected on kanamycin (50 μg/mL final) agar plates. Each scFv clone was expressed in 1 mL of kanamycin selective, auto-induction media [70] in a 96 deep well plate covered with a sheet of AirPore (Qiagen). Following over night (ON) incubation with shaking (1000 rpm) at 30°C, the expressed scFv protein was recovered from the media supernatant after spinning down the cells by centrifugation at 4000 rpm for 30 min. For screening, no further protein purification was required: 200 μl of supernatant was added to a 100 μl of PBS solution containing 10^6^-10^7^ washed bacteria cells and incubation was performed for 1 h at RT. Cells were washed twice with PBS and the scFv-GFP11 scFvs were fluorescently labeled using anti-SV5-IgG phycoerythrin conjugated antibody (anti-SV5-PE). After 1 h incubation at RT, cells were finally washed twice with PBS and analyzed using the HTS feature of the Becton Dickinson LSRII Flow Cytometer LSRII. The fluorescence data were collected using the high-throughput analysis feature of LSRII and analyzed by Flowjo (Tree Star, Inc.; Ashland, OR).

### Protein expression and purification

For larger scale production and purification, the anti*-Lactobacillus acidophilus* scFv (α-La) was expressed from the pEP-GFP11 plasmid but was scaled up to 2 L of auto-induction media. The culture grew at 37°C to mid-log phase then was shifted to 20°C ON (~16-20 hrs). Bacteria were harvested by centrifugation at 7000 rpm for 10 minutes and the cell pellet was stored at -80°C. Cell pellet was resuspended in lysis buffer consisting of 50 mM HEPES pH 7.3, 450 mM NaCl, 15 mM Imidazole, and 1 mg/ml lysozyme and after a brief incubation (30 minutes) on ice, further lysis was performed by means of a pressure press (EmulsiFlex–C5, Avestin Inc.). The bacterial debris was pelleted by centrifugation at 16,000 rpm for 30 minutes, and the soluble fraction was applied to Ni-NTA agarose resin (Qiagen Inc.). After incubation at 4°C for 30–60 minutes, the resin was spun down at 1000xg for 60s. The pelleted resin was added to an empty column and washed by gravity flow with copious amounts of lysis buffer. Protein was eluted off the Ni-NTA resin in a buffer containing 20 mM HEPES pH 7.3, 150 mM NaCl, and 300 mM Imidazole. Further purification was performed by Size Exclusion Chromatography (SEC) using a 320 ml Sephadex 200 column (GE lifesciences) in a buffer consisting of 20 mM HEPES 7.3, 150 mM NaCl, and 5% (v/v) glycerol. Fractions containing the scFv were pooled, aliquoted, flash frozen in liquid nitrogen, and stored at -80°C. Binding efficiency for flash frozen scFv versus unfrozen scFv were compared and the binding was identical (data not shown) demonstrating that the freezing the protein for long term storage did not alter binding capacity.

### Binding specificity assay

Purified, recombinant scFv was used to test specificity for *L. acidophilus*. Before the assay, the scFv was incubated with an excess of GFP1-10 complementary protein as described previously [37] ON at 4°C. The following day 5–15 μg of scFv with or without restored GFP were incubated with 10^6^-10^7^ bacteria in solution containing PBS and Wash Buffer (0.5% BSA, 2 mM EDTA). After 1 h incubation at RT the bacteria were washed twice with PBS and resuspended in a 1:1000–1:2000 anti-SV5-PE (1 μg/μl). Incubation was performed for 1 h at RT and the cells were washed and resuspended in PBS prior to analysis with two different flow cytometers. The BD LSRII was used to evaluate the mean average fluorescence for binding activity of the scFv, and the AMNIS was used to image fluorescently labeled scFv bound to cells. The same procedure was followed for the other *Lactobacillus* species and for the other species to clearly confirm the specificity of the scFv binding.

### Capture efficiency assay

Individual bacteria species (Table 1) were grown separately, washed, and all diluted in PBS to an OD_600_ of 1.0 where an absorbance of 1.0 is equal to ~10^9^ bacteria cells per milliliter. Equal volumes of each bacteria were mixed with *L. acidophilus* added at theoretical ratios of 10%, 5%, 1%, and 0.1%. α-La was prepared and incubated with bacterial mixtures as described above. Samples were analyzed on BD Influx. Three gates were used for the analysis: P1, P2, and P3. P1 was drawn to include bacteria defined by size and morphology using a two dimensional Side Scatter (SSC):Forward Scatter (FSC) plot. P2 and P3 are drawn in a two dimensional fluorescence (FITC:PE) plot and include bacteria captured in the P1 gate. P3 is drawn using a control sample consisting solely of *L. acidophilus* and therefore defines the region of the cytograph occupied by bacteria bound to PE and GFP 1–10 stained scFv. P2 represents bacteria in the culture that were not recognized by the scFv and are not fluorescent above background. In every experiment, stained and unstained versions of each sample are compared to ensure that there are no events in P3 for any of the unstained samples. We define the percent *L. acidophilus* in any sample as the number of events in P3 divided by the number of events in P1.

### Single cell sorting and sequencing from yogurt

Fresh yogurt was cultured from freeze-dried starter cultures (http://www.culturesforhealth.com) following manufacturer’s instructions. Bacteria were extracted from the yogurt within 24–48 hours of culturing as previously described [33], with modifications. Specifically, 20 g of yogurt from each independent yogurt culture was resuspended in 150 ml suspension solution in a Waring 34BL97 blender. After five cycles of 1-min blending at 17,000 rpm and 2-min incubation on ice, three 30 ml aliquots were made in 50 ml Falcon tubes. Eight milliliters of Nycoprep Universal 60% solution (Accurate Chemical; Westbury, NY) was directly injected to the bottom of the tube with a sterile syringe. A visible cell layer between the Nycodenz and aqueous layers was obtained by 2-hr centrifugation at 15,000 g at 4°C. Up to 3.5 ml of each cell layer was pooled in a 15 ml Falcon tube. After an initial centrifugation at 10,000 g for 15 min at 4°C was done, the cell pellet was washed by two cycles of centrifugation at 10,000 g for 15 min at 4°C, removal of supernatant, and resuspension in 1 ml sterile 1× PBS. 10^7^-10^8^ bacteria were set up in the binding assay with the α-La as described above. The resulting scFv-bound bacteria were analyzed and sorted using a BD Influx flow cytometer. The same three gates (P1, P2, and P3) were drawn as described for the mock community analysis but were used for sorting in this instance. Lab preparations, flow cytometer setup, MDA, and PCR steps were performed as previously described [24]. Briefly, 88 cells from each gate were single-sorted into discrete wells containing 2 μl lysis buffer of a 96-well PCR plate. For positive MDA controls, four wells received either 1 ng *E. coli* ATCC 29425 or *B. subtilis* ATCC 6633 purified DNA. The remaining four wells were no-template negative controls. After freeze-thaw lysing, MDA was performed at 16 hr and the products diluted at 1:100 in sterile water. One microliter of the diluted MDA product was used as template to generate ~1400 bp 16S rDNA PCR amplicons using 8 F (5′ – AGAGTTTGATCCTGGCTCAG) and 1492R (5′ – GGTTACCTTGTTACGACTT) primers. The PCR amplicons were purified (NucleoSpin 96 kit; Macherey Nagel, Germany) and Sanger-sequenced (ABI 3730) using the same PCR primers. Only contiguous sequences formed from both the forward and reverse reads were used in all analyses: Genus-level identification of sorted cells was done with RDP Classifier [71] under default settings, while species-level identification was done with Blastn. Statistical analysis and figure generation were performed using *R* (R Development Core Team). Confidence intervals (*CI*) were calculated using the formula: 95% *CI* = M ± (SE * 1.96) where M = Mean, SE = Standard Error.

### Genome sequencing

For the template-dependent genome comparison study, 50 cells or a single cell from the yogurt P3 gate were sorted into one PCR well each containing 2 μl lysis buffer, MDA-, and PCR-amplified, as described [24]. Blastn of the 16S rDNA PCR products from both the single cell and 50-cell templates showed >98% identity to *L. acidophilus* (NCFM). To compare genome coverage, the single- and 50-cell amplicons were sequenced using the Illumina MiSeq platform using standard Illumina libraries made using the TruSeq DNA Library prep kit. Sequencing data was normalized using equal numbers of reads from each sample followed by quality screening and trimming consisting of removal of ambiguous bases, ends trimmed with quality less than 10 and reads removed with average base-quality less than 20. Sequencing was performed using paired-end and non-paired end run resulting in ~151 bp reads with ~99% of the total reads being included after trimming. Reads were mapped to the *L. acidophilus* (NCFM) reference using the CLC Genomics Workbench (CLC bio). 83.9% and 88.2% of the single-cell and 50-cell (respectively) reads were mapped to the reference resulting in 68.6% and 99.9% coverage of the reference genome. The single-cell or 50-cell data resulted in 516 or 12 gaps with gap lengths ranging from 1 to 26,493 bps for the single cell and 3 to 862 bp for the 50-cell data. For *de novo* assembly, prior to contaminant removal the sequencing data from the 50 cell template assembled into 2,931 contigs with N50 equal to 5,811 bp and minimum contig length of 177 bp with the longest contig being 157,137 bp long. The single cell sequence data assembled into 595 contigs with N50 equal to 7,100 bp with the minimum contig length equal to 200 bp and the longest contig being 62,621 bp. After removal of contaminants, *de novo* assembly using CLC resulting in 555 contigs (from the single cell assembly) or 124 (from the 50 cell assembly) and were mapped back to the reference to assess coverage. Figures were generated using *R* as described above.

### Western blot and antigen identification by mass spectrometry

Bacteria (10^10^) were lysed by resuspending the cells in a SDS-PAGE lysis buffer containing 2% SDS and 0.6 M β-mercaptoethanol and boiling at 98°C for 15 minutes. The lysed sample was run on a 4-12% SDS-PAGE gel and the separated protein was subsequently transferred to nitrocellulose membrane for Western Blot. The membrane was blocked in Casein blocking solution (Thermo Scientific) followed by incubation with 0.5 ug/ml recombinant α-La scFv in PBS for 1–2 hrs at RT. Following incubation with α-La scFv, the membrane was washed 1× with PBST followed by two washes with PBS, then incubated with 1:1000 dilution of anti-SV5 IgG conjugated to Alkaline Phosphatase (AP). The blot was developed using 1-step NBT/BCIP (Thermo Scientific). A single band corresponding to a molecular weight of ~45 KDa was observed in the western blot. The band was cut out and washed thoroughly with water in a 1.5 ml centrifuge tube. Extracted bands from the Western Blot were subjected to trypsin (2 ng and 20 ng Trypsin Gold, Promega, Madison, WI) digestion overnight at 37°C. The resultant peptides were analyzed by MALDI-TOF/TOF on a 4800 Plus (AB Sciex, Foster City, CA) using standard methods for peptide MS and MS/MS. The MS/MS data were analyzed using ProteinPilot Software version 4.0 against a *L. acidophilus* NCFM fasta database using a 95% confidence level threshold. The peaks matched two peptide sequences (SATLPVVVTVPNVAEPTVASVSKR and IMHNAYYYDKDAKR), both mapping to the S-layer A protein (SlpA), from *L. acidophilus* with >95% confidence. To test if glycosylation was important for binding, *L. acidophilus* was deglycosylated using a mixture of enzymes containing PNGase F, O-Glycosidase, Neuraminidase, β-1,4 Galactosidase, and β-N-acetylglucosaminidase (New England Biolabs).

### Deep sequencing of HCDRs

Eighteen antibody framework 3 VH specific primer pairs have been used to amplify the HCDR3 portion of the scFvs. The amplicons have been sequenced on Ion Torrent using the Ion 316 Chip kit by the recommended standard protocol. The Ion Torrent outputs have been analyzed by the Antibody Mining ToolBox software package (http://sourceforge.net/projects/abmining[50]) using the default quality trimming values. The resulting HCDR3 abundance files were imported into spreadsheet software for further analysis.

### Data deposition

The *Lactobacillus acidophilus* genomes assembled from single cell or 50-cell templates were deposited in the NCBI database under the Assembly names L acidophilus CFH 1_cell and L acidophilus CFH 50_cells. The BioSample, Genome Accession, and Raw Data File numbers are: SAMN02401338, AYUA00000000, SRR1029918 for the 1_cell assembly and SAMN02401339, AYUB00000000, SRR1029904 for the 50_cells assembly.

## Abbreviations

NGS: Next generation sequencing; SCG: Single cell genomics; MDA: Multiple displacement amplification; scFv: Single chain variable fragment; FACS: Fluorescence activated cell sorting; SlpA: S-layer A protein; GFP: Green fluorescent protein; PE: Phycoerythrin; HCDR3: Heavy chain complementarity determining region 3.

## Competing interests

The authors declare no competing financial interests.

## Authors’ contributions

DC and FF planned the experiments, carried out the phage selection and the molecular studies, participated in sorting experiments, and drafted the paper. NV and SK participated in the phage selection. AEKD carried out the sorting experiment with KR and supervised the genomic analysis conducted by ARD. HD performed the statistical analysis. TCS and SI carried out the antigen identification by mass-spectrometry. CK and SK performed the deep sequencing analysis of the HCDR3. CSH and ARMB conceived the study, and participated in its design and coordination and helped to draft the manuscript. All authors read and approved the final manuscript.

## Supplementary Material

Additional file 1 **Sequence alignment of the four scFvs selected against *L. acidophilus.*** HCDR3 sequences are highlighted in yellow. Click here for file

Additional file 2 **Binding of the four unique anti-La scFvs to different Lactobacillus species using scFv culture supernatant and flow cytometry.** The anti-La scFvs are all specific to *L. acidophilus* and the anti-La2 may discriminate between *L. acidophilus* strains. Click here for file

Additional file 3 **Bacteria identified in various gates after single cell sorting and classification.** Approximately 88 cells were sorted from each gate for each replicate. Species identities reported at >94% maximum identity by Blastn search of the 16S rDNA sequences. Replicates are different bacteria preps isolated from yogurt cultures and the gates correspond to gates shown in Figure 4 of the main text. Click here for file
