# Supplementary material for: Using phage display selected antibodies to dissect microbiomes for complete de novo genome sequencing of low abundance microbes
Source: BMC Microbiol. 2013 Nov 27;13:270. doi: 10.1186/1471-2180-13-270 (PMC3907030; doi:10.1186/1471-2180-13-270)
Supplement: Additional file 1 — Sequence alignment of the four scFvs selected against L. acidophilus. HCDR3 sequences are highlighted in yellow. [file 1471-2180-13-270-S1.pdf]

Additional File 1. Sequence alignment of the four scFvs selected against *L. acidophilus*. HCDR3 sequences are highlighted in yellow.

```
α -La1        GAHASYELTQPPSASGTPGQRVTISCSGSSSNIGS-NYVYWYQQLPGTAPKLLIYRNNQR 59
α -La2        GAHAQSVLTQPASVSGSPGQSITISCTGTSSDVGSYNYVSWYQQHPGKAPKLMIYDVSNR 60
α -La3        GAHASYVLTQPPSVSGTPGQRVTISCSGSSSNIER-NYVYWYQQLPGTAPKLLIYRNNQR 59
α -La4        GAHAQAVVTQEPSASETPGQRVTISCSGSDSNIGS-NYVYWYQQVPGTAPKLVIYRNNQR 59
              ****.  :** .*.* :*** :****:*:.*::   *** **** **.****:**  .:*

α -La1        PSGVPDRFSGSKSGTSASLAITGLQAEDEADYYCQSYDSSLSGYVFGTRTKLTVLSGGST 119
α -La2        PSGVSNRFSGSKSGNTASLTISGLQAEDEADYYCSSYTSS-STNVFGTGTQLTVLSGGST 119
α -La3        PSGVPDRFSGSKSGTSASLAISGLRSEDEADYYCAAWDDSQNAYVFGTGTKLTVLSGGST 119
α -La4        PSGVPDRFSGSKSGTSASLGIRGLQSEDEADYYCAAWDDSVSGWVFGGRTKLTVLSGGST 119
              ****.:********.:*** * **::******** :: .* .  ***  *:*********

α -La1        ITSYNVYDTKLSSSGTQVQLQQSGPGLVKPSQTLSLACAISGDSVSSNSAAWNWIRQSPS 179
α -La2        ITSSNVYYTKLSSSGTEVQLVETGGGLVKPGGSLRLSCAASGFTLSN--AWMHWVRQAPG 177
α -La3        ITSYNVYYTKLSSSGTQVQLQESGPGLVKPSETLSLTCAISGDSVSSNSAAWNWIRQSPS 179
α -La4        ITSYNVYYTKLSSSGTQVQLQQSGPGLVKPSQILSLTCAISGDSVSSNSADWNWIRQSPS 179
              *** *** ********:*** ::* *****.  * *:** ** ::*.  *  :*:**:*.

α -La1        RGLEWLGRIYYRSK-WYNDYAVSVKSRITINPDTSKNQFSLQLNSVTPEDTAVYYCARER 238
α -La2        KGLEWVGRIRRKTDGGTTDYAAPVKGRFTISRDDSKNTLYLQMNSLKTEDTAVYYCSTD- 236
α -La3        RGLEWLGRTYYRSK-WYNDYAVSVKSRITINPDTSKNQFSLQLNSVTPEDTAVYYCATG- 237
α -La4        RGLEWLGRTYYRSK-WYNDYAVLVKSRITINPDTSKNQFSLQLNSVTPEDTAVYYCARGS 238
              :****:**   ::.   .***. **.*:**. * *** : **:**:..********:

α -La1        YGAFDIWGQGTMVTVSSASGKPIPNPLLGLDST 271
α -La2        -DYGGNWGQGTLVTVSSASGKPIPNPLLGLDST 268
α -La3        -DAFDMWGQGTMVTVSSASGKPIPNPLLGLDST 269
α -La4        LGAFDIWGQGTMVTVSSASGKPIPNPLLGLDST 271
               .  . *****:*********************
```
