# Supplementary material for: Using phage display selected antibodies to dissect microbiomes for complete de novo genome sequencing of low abundance microbes
Source: BMC Microbiol. 2013 Nov 27;13:270. doi: 10.1186/1471-2180-13-270 (PMC3907030; doi:10.1186/1471-2180-13-270)
Supplement: Additional file 2 — Binding of the four unique anti-La scFvs to different Lactobacillus species using scFv culture supernatant and flow cytometry. The anti-La scFvs are all specific to L. acidophilus and the anti-La2 may discriminate between L. acidophilus strains. [file 1471-2180-13-270-S2.pdf]

Additional File 2

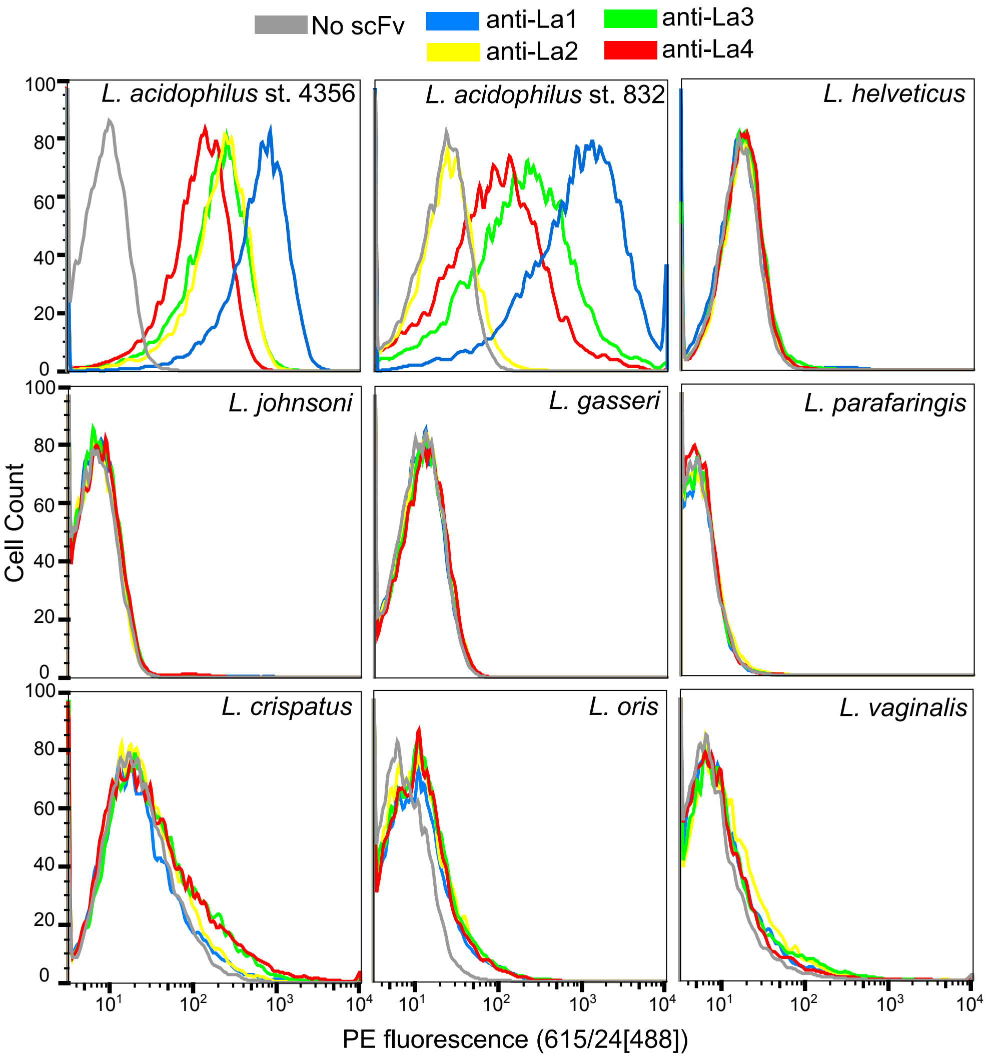

Binding of the four unique anti-La scFvs to different Lactobacillus species using scFv culture supernatant and flow cytometry. The anti-La scFvs are all specific to *L. acidophilus* and the anti-La2 may discriminate between *L. acidophilus* strains.
