# Supplementary material for: Using phage display selected antibodies to dissect microbiomes for complete de novo genome sequencing of low abundance microbes
Source: BMC Microbiol. 2013 Nov 27;13:270. doi: 10.1186/1471-2180-13-270 (PMC3907030; doi:10.1186/1471-2180-13-270)
Supplement: Additional file 3 — Bacteria identified in various gates after single cell sorting and classification. Approximately 88 cells were sorted from each gate for each replicate. Species identities reported at >94% maximum identity by Blastn search of the 16S rDNA sequences. Replicates are different bacteria preps isolated from yogurt cultures and the gates correspond to gates shown in Figure 4 of the main text. [file 1471-2180-13-270-S3.pdf]

Additional File 3

Replicate	Gate	Bacterial Isolates Identified After Sorting											Total
		Streptococcus sp.	*Rhodococcus* sp.	*Dietzia* sp.	*Bradyrhizobium* sp.	*Escherichia* or *Shigella*	*Lactobacillus acidophilus*	*Lactobacillus delbrueckii*	*Corynebacterium* sp.	*Rhizobium* sp.	*Bacillus* sp.	*Variovorax* sp.	
**A**	**P1**	18	4	0	0	1	4	1	0	0	0	1	29
	**P2**	18	1	0	0	0	0	2	1	0	0	0	22
	**P3**	2	2	1	1	0	17	1	0	0	0	0	24
**B**	**P1**	23	0	0	0	0	0	2	0	0	1	0	26
	**P2**	24	0	0	0	0	0	0	0	0	0	0	24
	**P3**	0	0	0	0	0	4	0	0	0	0	0	4
**C**	**P1**	17	0	0	0	0	0	2	0	0	0	0	19
	**P2**	13	0	0	0	0	0	0	0	0	0	0	13
	**P3**	0	0	0	0	0	6	0	0	0	0	0	6
**D**	**P1**	28	0	0	0	0	0	1	0	1	0	0	30
	**P2**	35	0	0	0	0	0	0	0	0	0	0	35
	**P3**	1	0	0	0	0	11	0	0	0	0	0	12

Bacteria identified in various gates after single cell sorting and classification. Approximately 88 cells were sorted from each gate for each replicate. Species identities reported at >94% maximum identity by Blastn search of the 16S rDNA sequences. Replicates are different bacteria preps isolated from yogurt cultures and the gates correspond to gates shown in Fig. 4 of the main text.
